# Phase-Field Simulation of Grain Boundary Evolution In Microstructures Containing Second-Phase Particles with Heterogeneous Thermal Properties

**DOI:** 10.1038/s41598-019-54883-8

**Published:** 2019-12-05

**Authors:** T. F. Flint, Y. L. Sun, Q. Xiong, M. C. Smith, J. A. Francis

**Affiliations:** Dalton Nuclear Institute, The University of Manchester, Manchester, M13 9PL UK

**Keywords:** Computational science, Computational methods, Surfaces, interfaces and thin films, Applied mathematics, Coarse-grained models

## Abstract

Understanding the interaction between complex thermal fields and metallic structures at the meso-scale is crucial for the prediction of microstructural evolution during thermomechanical processing. The competitive growth of crystal grains, driven by thermodynamic forces at the grain boundaries, is one of the most fundamental phenomena in metallurgy and solid state physics. The presence of second phase particles, which act as pinning sites for boundaries, drastically alters the coarsening behaviour of the system; particularly when considering that these particles have different thermal properties to the primary phase. In this work a multi-phase field model, incorporating thermal gradient and curvature driving forces, is used to predict grain growth in a *Ti6Al4V* alloy system with second phase particle inclusions representative of oxide and carbide precipitates. The multi-phase field framework is fully coupled to the heat equation. The incorporation of the thermal gradient driving force enables the detailed behaviour of the grain boundaries around the particles to be predicted. It is shown that the inclusion of particles with a lower thermal conductivity has a significant influence on the coarsening behaviour of various systems of grains, due to the combined effects of thermal shielding and the generation of thermal gradient driving forces between the boundaries and pinning particles.

## Introduction

The application of intense sources of heat to a poly-crystalline system generates large internal thermal gradients resulting in highly heterogeneous microstructures^1^. The final microstructure largely determines the physical properties and it may favourably or adversely affect material performance. The modelling of microstructual evolution is therefore of great academic and industrial interest^2,3^. In a cold worked or heavily deformed metal or alloy, a thermal cycle may lead to a number of processes taking place, such as recovery, recrystallisation and/or grain growth. There are many situations in which grain growth alone occurs such as, for example, in fusion welding. In this work, we focus on a method for the faithful modelling of grain growth.

Grain boundaries migrate in response to driving forces acting upon them, in such a manner as to minimise the total free energy in the system^4^. Second phase particles present within the microstructure strongly influence the boundary migration kinetics due to their capacity to ‘pin’ grain boundaries^5,6^. New boundaries must be created to traverse the particles; this boundary creation has an an energy cost to the system^7^. The exact composition of these second phase precipitates is determined by the chemical composition of the alloy, and current thermodynamic state of the system. A common family of precipitates, and inclusions, that are observed in many metallic systems however are those rich in elements with lower thermal conductivity (*k*) such as Si, O and C which may form oxide, carbide and silicate type precipitates^8–12^.

It is known that strong thermal gradients drive extended defects, such as grain boundaries and voids, to migrate in preferential directions^13–15^. Molecular dynamics simulations have been conducted to study thermal gradient driven grain boundary migration and verify a previously proposed thermal gradient driving force description^13^. Recently, the combined effects of thermal-gradient and curvature driving forces on the microstructural evolution have been studied for an isotropic microstructure; it has been shown that the thermal gradient driving force is an important factor in determining the coarsening behaviour in the vicinity of high energy density sources of heat, such as electron-beam, electric-arc and laser welding heat sources, all of which are utilised in advanced manufacturing processes^2^. Given that the temperature field induced in a material is the primary mechanism by which competitive crystal growth is initiated, and that the thermal gradient driving force is an important contribution in determining boundary migration kinetics, it is clear that second phase particles present in the microstructure with heterogeneous thermal properties relative to the primary phase will strongly perturb the induced local temperature field and generate additional thermal gradients influencing the microstructural evolution behaviour.

Consideration of the interaction between the temperature field generated due to heterogeneous thermal properties on the meso-scale and grain boundary migration enables new physical insights to be obtained on the evolution of grain sizes. In prevalent analytical models of grain growth, the effects of pinning particles are considered solely as the source of a dragging force which uniformly applies to all the growing grains^16–18^. Such a modelling approach is convenient to estimate the average grain size, but it cannot reveal the role that second phase particles play in the change of thermal condition which affects the grain growth, particularly when the temperature gradient is high and the associated driving energy is significant. In order to capture the physical mechanisms that result in the coarsening of microstructures an accurate mathematical model is required. Several techniques for the simulation of grain growth exist. Monte Carlo simulations based on the Potts model^19–21^, molecular dynamics simulations^22^, cellular automata simulations^23^, and the phase field method^4,24^ are all used to determine the evolution behaviour of microstructures over various length and time scales. The phase field method is generally considered to be the most versatile, mature, and physically representative of the system over realistic time scales, despite being one of the more computationally expensive of the methods^1^. The phase field model permits thermodynamic driving forces to be incorporated into the free energy functional of the system to obtain realistic evolution dynamics^25^. Many driving forces can be considered in this framework, however the dominant driving force considered during the evolution of grain boundary networks in stress free scenarios is the curvature driving force. In order for the thermal gradient driving force to be significant, the magnitude of the thermal gradient must be sufficiently high. For this reason, the thermal gradient driving force is typically omitted in the phase field representation of the system. However, in advanced manufacturing processes where the thermal gradient magnitude can be of the order of $\sim {10}^{7}\,K\,{m}^{-1}$ in the vicinity of the heat source, the thermal gradient driving force becomes important. Large thermal gradients in a material lead to the development of large thermal stresses, and additional mechanical driving forces for grain boundary evolution due to elastic deformation^26,27^. Simplified approaches for incorporating the effects of second phase particles in larger length scale simulations also exist, however these approaches, which generally consider different thermal activation energies for grain structures with the presence and absence of second phase particles, do not explicitly consider the particles, and consequently the perturbation of the thermal field due to the particles is not captured^28^.

In this work a multi-phase field formulation for the prediction of grain coarsening due to local boundary curvature and thermal gradient driving forces, is fully coupled to the heat equation. This framework permits the prediction of grain boundary evolution, in the vicinity of high energy density sources of heat, with various distributions of second phase particles with homogeneous and heterogeneous thermal properties relative to the primary metallic phase. In this study the particles are assumed to be spatially and temporally static, and the mechanical driving forces are neglected as the focus of the investigation is to determine the importance of purely thermal effects on the microstructural evolution. Various scenarios are investigated using the model, with key insights gleaned from the framework regarding the migration kinetics of single grain boundaries, and grain boundary networks, over second phase particles with heterogeneous thermal properties.

## Results

Three scenarios are investigated using the mathematical framework described. Initially the behaviour of a single grain boundary, migrating over a particle, is investigated. The model is then applied to grain boundary networks in isothermal conditions. Finally the framework is used to investigate the response of a microstructure due to the application of a rapid heating and cooling cycle representative of those observed in advanced manufacturing processes. For generality, the heterogeneous thermal conductivity of the particles, *k*, in this work is conservatively estimated, from experimental measurements of various oxide and carbide conductivities to be 1/20 of the parent conductivity. The specific heat, *c*_*p*_, and density, $\rho$, of the particles is assumed to be equal to that of the grains. In reality the thermal conductivity of certain common second phases is known to be considerably lower.

### Migration behaviour of a single boundary around a second phase particle

**Figure 1:**
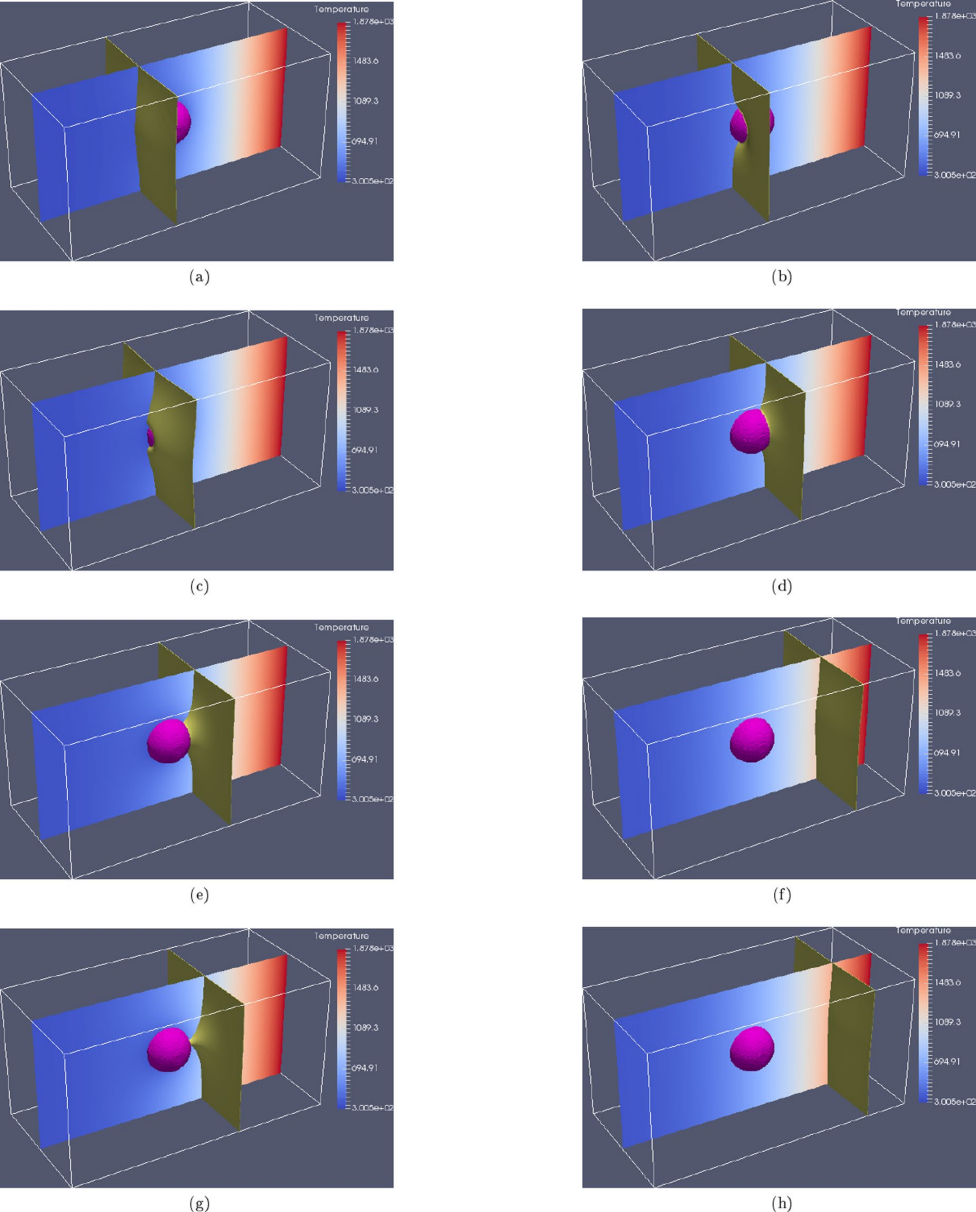
Migration of a single grain boundary around a second phase particle for two scenarios with the same thermal load applied at the right hand side of the domain; Case 1 where the particle has a lower thermal conductivity than the grains (${k}_{particle} < {k}_{grains}$) is shown in (**a**,**c**,**e**,**g**), while Case 2 where the thermal conductivity of the particle is equal to that of the grains (${k}_{particle}={k}_{grains}$) is shown in (**b**,**d**,**f**,**h**) at times of $2.05\times {10}^{-3}\,s$, $2.44\times {10}^{-3}\,s$, $2.85\times {10}^{-3}\,s$ and $2.92\times {10}^{-3}\,s$ respectiveley.

In the first instance, the behaviour of a single grain boundary in a time varying temperature field is investigated. A three dimensional domain of 0.5 *mm* by 0.25 *mm* by 0.25 *mm* was generated and a spherical particle of radius 4 × 10^−2^ *mm* was placed in the domain, centred at $(0.25\,mm,0.125\,mm,0.125\,mm)$. Two grains were also placed in the domain such that a grain boundary existed in the *y*–*z* plane at $x=0.166\,mm$. At $t=0\,s$ the temperature throughout the domain was initialised to 300 *K*. The $x=0.5\,mm$ face of the computational domain was then set, and maintained, at *T*_*solidus*_. The temperature evolution within the domain is governed by Eq. 6. Two cases were investigated; one in which the thermal conductivity of the particle is lower than that of the grains, *Case 1*, and one in which the thermal conductivity of the particle was equal to that of the grains, *Case 2*, as shown in Fig. 1.

During the initial stages of the simulation there is negligible thermal gradient in the domain, and no curvature driving force acting on the boundary and so it remains stationary. As the temperature field evolves, a temperature gradient is generated throughout the domain, exerting a driving force. In *Case 1*, where the thermal conductivity of the particle is lower, it takes longer for this driving force to reach a sufficient magnitude to initiate movement as the pinning particle with lower thermal conductivity shields the boundary. Following movement initiation up the temperature gradient, as the boundary makes contact with the particle, the relatively large particle curvature initially attracts the boundary, as shown in Fig. 1(a) for *Case 1* and Fig. 1(b) for *Case 2*. The particle pinning effect then becomes dominant as new boundary is created during the traverse, retarding the progression of the boundary up the thermal gradient as shown in Fig. 1(e) for *Case 1*, until the boundary curvature force is sufficient to overcome the pinning effect.

**Figure 2:**
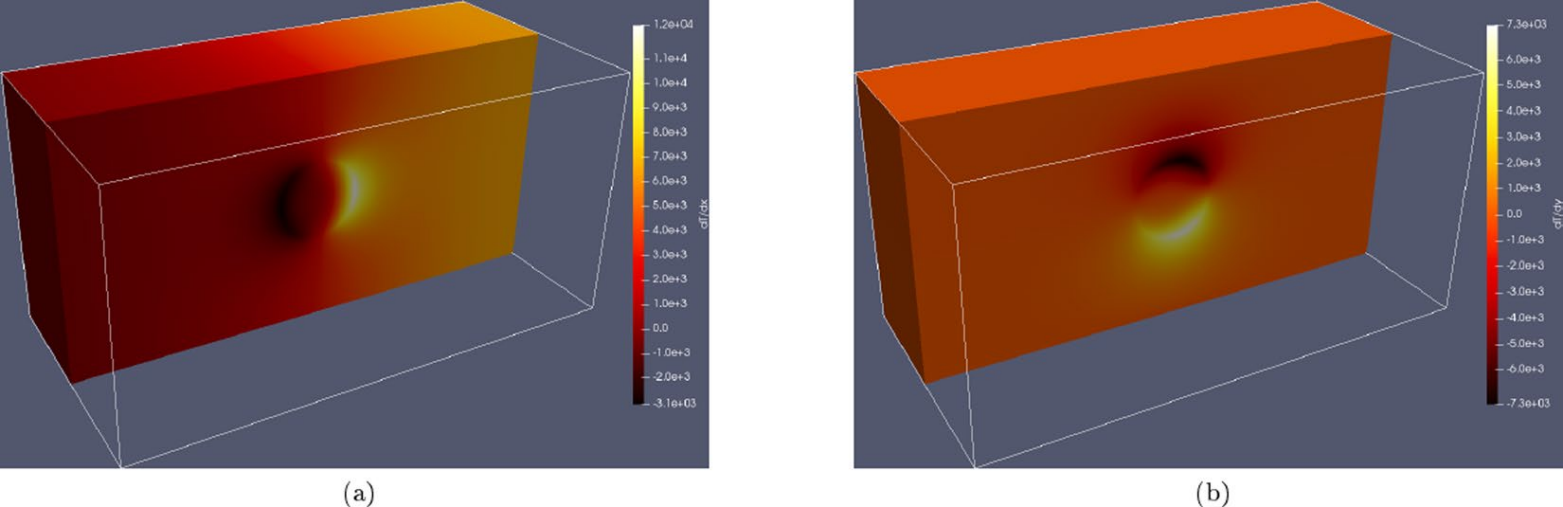
Temperature gradient components around the second phase particle at $2.85\times {10}^{-3}\,s$; (**a**) shows $\partial T\partial x$ and (**b**) shows $\partial T\partial y$ around a second phase pinning particle with lower thermal conductivity than the primary phase. Non-trivial thermal gradient driving forces are generated on the grain boundary as it approaches and migrates over the particle.

In *Case 2*, the thermal gradient is simply normal to the *x*–*y* plane. In *Case 1* however, a thermal gradient is generated around the particle due to the varied thermal properties and so the thermal gradient driving force on the boundary is non-trivial, as shown in Fig. 2.

The net effect of the presence of a second phase particle with lower thermal conductivity is to perturb the heat propagation through the domain, effectively shielding the boundary on the other side of the particle and requiring longer for the driving force in the vicinity of the boundary to be sufficient for migration. Additional components of the thermal gradient driving force are also generated, further influencing the boundary during the traverse. The overall effect is an increase in the time taken for the boundary to traverse the particle, which is analogous to an increase in the amount of energy required to traverse the particle.

### On the effect of particle size distributions in an isothermal domain

**Figure 3:**
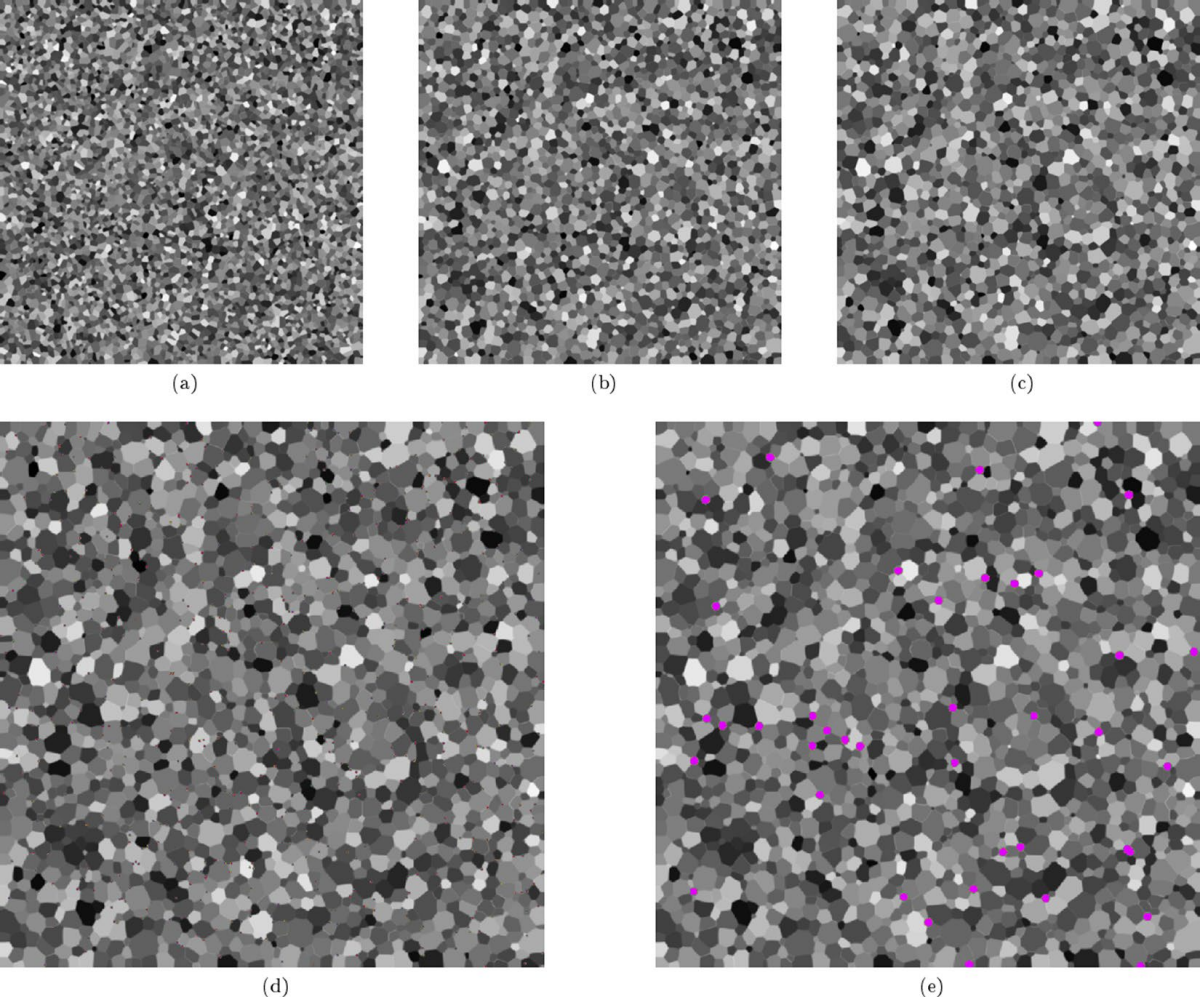
Evolution of the 2D simulation domain for the isothermal scenario where three particle distributions are considered. (**a**–**c**) Shows the evolution of the domain with no second phase particles present at $t=1.6\times {10}^{-4}\,s$, $t=3.2\times {10}^{-3}\,s$ and $t=6.4\times {10}^{-3}\,s$ respectiveley. (**d**) Shows the domain with 640 particles, each with a radius ${r}_{p}=4\,\mu m$ at $t=6.4\times {10}^{-3}\,s$. (**e**) Shows the domain with 40 particles, each with a radius ${r}_{p}=16\,\mu m$ at $t=6.4\times {10}^{-3}\,s$. Particles are shown in magenta with grains coloured based on phase field.

**Figure 4:**
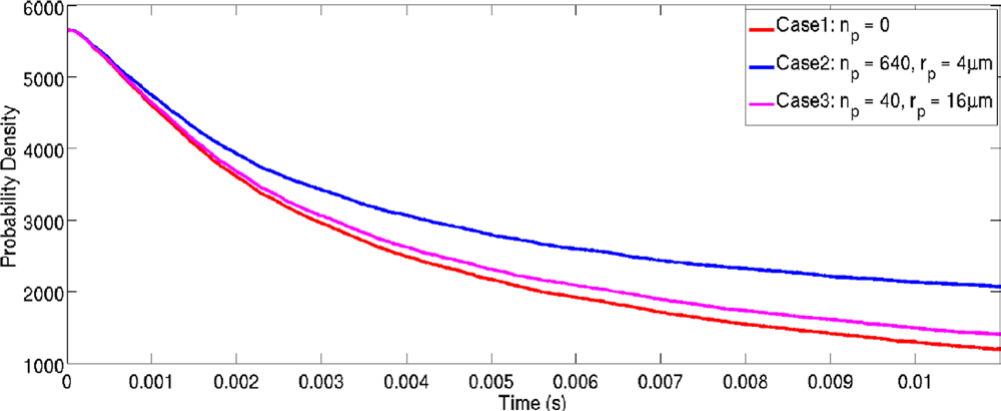
Time Evolution of the number of grains in the simulation domain for the isothermal domain case with different pinning particle distributions.

The effect of pinning particle size on the grain boundary evolution behaviour of a *2D* domain was investigated for an isothermal case. In the presented cases a 2 *mm* by 2 *mm* domain was initialised with $N=5656$ grains, corresponding to an average equivalent grain radius of 15 *μm* for the initial microstructure. The temperature of the domain was maintained at the solidus temperature of the alloy system (1878 *K*). The computational domain was discretised into 504.2 × 10^3^ grid points with $\Delta x=\Delta y=2.82\,\mu m$. The time discretisation, was performed with $\Delta t=1\times {10}^{-6}\,s$. Three cases were investigated: (*1*) a case where no pinning particles were present in the microstructure, (*2*) a case where 640 second phase particles were present in the domain with a radius of $4.0\times {10}^{-6}\,m$ each, and (*3*) a case where 40 second phase particles were present in the domain with a radius of $16.0\times {10}^{-6}\,m$ each. In the two cases where pinning particles are present the area fraction of particles in the domain is therefore equal. A time-step of $1.6\times {10}^{-6}\,s$ and a uniform grid spacing of 2 *μm* were chosen for the simulation. Figure 3 shows snapshots of the grain boundary network in each of the cases, and Fig. 4 shows a plot of grain number as a function of time.

As can be seen from Fig. 4, as the time progresses the number of grains decreases. As one would expect, the strongest pinning effect is for the case with a finer distribution of particles, *Case 2*, as the surface area of these particles is greater than for *Case 3*. For both cases where pinning particles are present in the matrix the number of grains in the domain is stabilised between *6* *ms* and *10* *ms*, the change in the number of the grains in the domain after this time is minimal and the domain is in equilibrium. In this isothermal scenario, clearly the dominant effect in determining the grain size and grain size distribution is the particle size.

### The effect of particle size distributions in a domain experiencing complex thermal load

**Figure 5:**
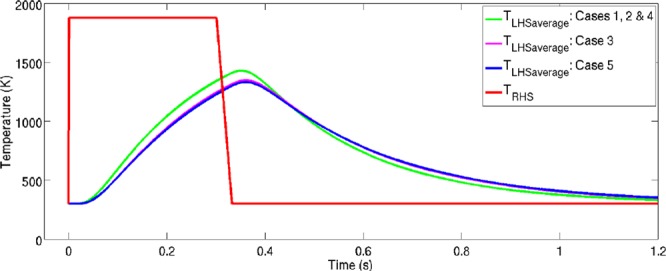
Average Thermal Response on the left and right of the Domain due to Various Particle Conductivities and Size Distributions.

**Figure 6:**
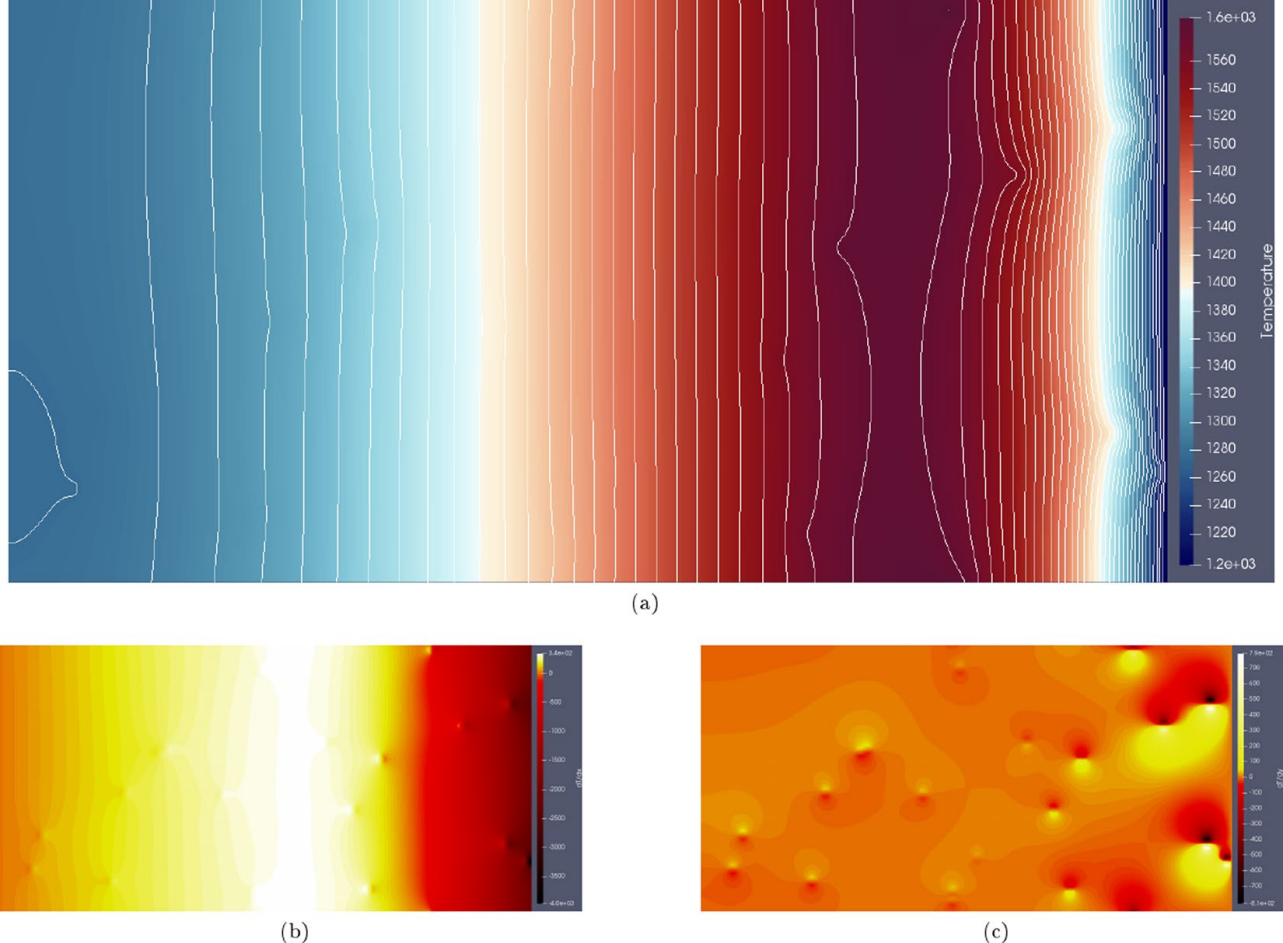
Perturbation of the temperature field (**a**), $\partial T\partial x$ (**b**) and $\partial T\partial y$ (**c**) within the domain due to the inclusion of second phase particles with a lower thermal conductivity than the primary phase at $t=0.314\,s$.

The effect of a non-trivial thermal load on a 2D domain was also investigated. In the following simulations a 2 *mm* × 1 *mm* domain was initialised with 2828 grains, again corresponding to an average equivalent grain radius of 15 *μm* for the initial microstructure as with the previous scenario. Five cases were considered: (1) No particles are present in the domain, (2) 320 Particles with $r=4\times {10}^{-6}\,m$ and $k=7.3\times {10}^{3}\,kg\,mm\,{s}^{-3}\,{K}^{-1}$, (3) 320 Particles with $r=4\times {10}^{-6}\,m$ and $k=0.365\times {10}^{3}\,kg\,mm\,{s}^{-3}\,{K}^{-1}$, (4) 20 Particles with $r=16\times {10}^{-6}\,m$ and $k=7.3\times {10}^{3}\,kg\,mm\,{s}^{-3}\,{K}^{-1}$, (5) 20 Particles with $r=16\times {10}^{-6}\,m$ and $k=0.365\times {10}^{3}\,kg\,mm\,{s}^{-3}\,{K}^{-1}$. A time dependent temperature was applied on the right hand side of the domain ($x=2\,mm$). This temperature was set to rapidly increase from ambient temperature to *T*_*solidus*_, remaining at the solidus temperature for 0.3 *s*, before cooling to ambient temperature. This thermal history is intended to be representative of that experienced in a welding heat affected zone. This applied temperature is shown in Fig. 5. The computational domain was discretised into 252.1 × 10^3^ points with $\Delta x=\Delta y=2.82\,\mu m$. The time discretisation, limited by the solution of the heat equation, was performed with $\Delta t=2\times {10}^{-7}\,s$. Figure 6 shows the temperature field and temperature gradient components due to the inclusion of particles, with a lower thermal conductivity than the primary phase and $r=16\times {10}^{-6}\,m$ (Case 5).

It is clear from Fig. 6 that the inclusion of the particles with lower *α* has a considerable effect on the temperature field. The generation of thermal gradients in the domain due to the particles influences the boundaries around these particles due to the thermal gradient driving force in the phase field equations. The grain boundaries migrate up the thermal gradient; therefore on cooling, where the particles remain at a higher temperature than their immediate surrounding primary phase due to their lower *α*, there is a strong thermal gradient driving force acting to pull the boundaries towards these particles. Figure 5 shows the average temperature response at the left hand side of the domain for the 5 cases.

**Figure 7:**
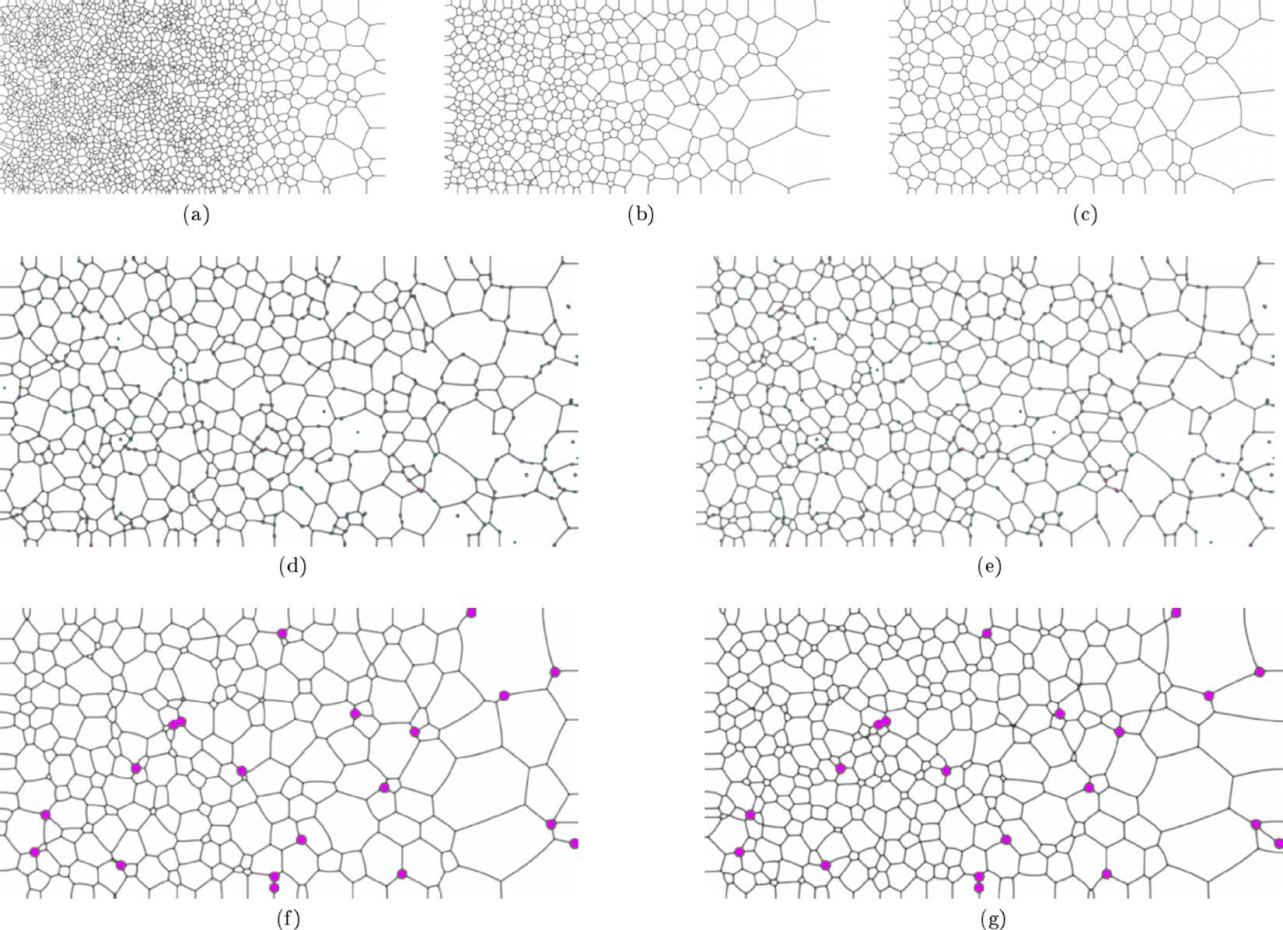
Grain Boundary Evolution for the 5 Cases due to Heating Impulse Shown in Fig. 5. (**a**–**c**) Show the grain structure evolution with no particles present (Case 1) at $t=0.151\,s$, $t=0.301\,s$ and $t=0.451\,s$ respectiveley. (**d**) Shows the final grain structure at $t=0.451\,s$ for the case where 320 particles, each with ${r}_{p}=4\,\mu m$, and ${k}_{particles}={k}_{grains}$ (Case 2). (**e**) Shows the final grain structure at $t=0.451\,s$ for the case where 320 particles, each with ${r}_{p}=4\,\mu m$, and ${k}_{particles}\ne {k}_{grains}$ (Case 3). (**f**) Shows the final grain structure at $t=0.451\,s$ for the case where 20 particles, each with ${r}_{p}=16\,\mu m$, and ${k}_{particles}={k}_{grains}$ (Case 4). (**g**) Shows the final grain structure at $t=0.451\,s$ for the case where 20 particles, each with ${r}_{p}=16\,\mu m$, and ${k}_{particles}\ne {k}_{grains}$ (Case 5). Second phase particles are shown in magenta.

As expected the peak average temperature experienced at the left hand side of the domain for cases 3 and 5 is lower than that for cases 1, 2 and 4, where there are no inclusions with a lower *α*. Similarly, upon cooling the uniform conductivity cases cool down more rapidly than cases 3 and 5. The variation in the thermal responses between cases 3 and 5 are due to the variation in spatial distribution of the particles, and the effect of a larger heat sink capacity of the larger particles. Figure 7 shows case 1 at three times (*0*.*151* *s*, *0*.*301* *s and 0*.*541* *s*) and the remaining cases at *0*.*451* *s*. This thermal shielding effect of the lower *α* particles dampens the propagation of the heat flux through the domain. The evolution history for *Case 2* can be viewed in the supplementary movie accompanying this work.

**Figure 8:**
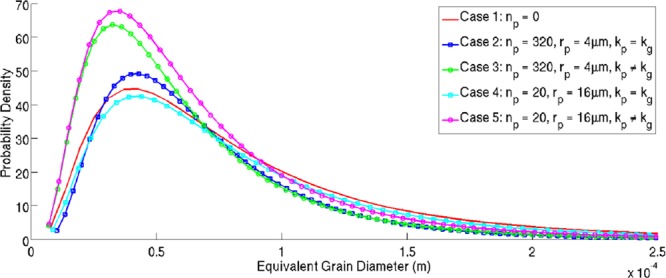
Grain Size Distributions for the 5 Cases Presented in a Domain Experiencing a Temperature Impulse.

As can be seen in Fig. 7, there has been increased coarsening towards the left hand side of the domain in cases 1, 2 and 4. In case 1 this is explained by the lack of pinning particles. Comparing the cases with the same particle distributions (case 2 to case 3, and case 4 to case 5) it is clear again that an increased degree of coarsening has occurred on the left hand side of the domain where the particles have the same conductivity as the primary phase. Generally speaking, the largest grains present at the end of the simulation are not as strongly influenced by the thermal properties of the particles but rather the particle distribution. Figure 8 shows the grain size distributions for the 5 cases, with circular markers representing the cases with heterogeneous thermal properties of the grains and particles, and square markers representing homogeneous thermal properties of the grains and particles.

Figure 8 clearly shows the effect of thermal shielding, and additional thermal gradient generation, due to the second phase particles being of a lower thermal conductivity. Cases 3 and 5 display a clear shift towards smaller grain diameters. It is interesting to note that the shift in the grain size distributions due to the heterogeneity in *α* is greater than the grain size shift due to the variation in particle size between the ${n}_{p}=320$ and ${n}_{p}=320$ cases. Considering that in isothermal, or near isothermal, cases it is assumed that the second phase particle size, and distribution, are the dominating factors determining grain size distribution.

## Discussion

In this work the grain boundary evolution kinetics of a poly-crystalline *Ti6Al4V* metallic matrix, containing second phase particles, is studied using a multi-phase field model incorporating thermal gradient and curvature driving forces. Mechanical driving forces on grain boundaries, induced by thermal strains, are typically the dominant forces determining boundary migration in the scenarios presented. However, as the effect of heterogeneous thermal properties has not been studied previously, in the in the present study the mechanical driving forces are neglected to systematically determine the effect of the heterogeneous thermal properties, and the thermal gradients these induce, on the microstructural evolution in heat affected zones. The effects of heterogeneous thermal properties between the particles and the matrix are studied through fully coupling the phase field equations to the heat equation. The insights gained from the model are highly relevant to the understanding of microstructural evolution in the vicinity of high energy density heat sources, as are experienced in heat affected zones during advanced manufacturing processes, where oxide and carbide type precipitates may form with lower thermal diffusivities than the primary phase. Fully understanding the effect of the precipitate distributions and thermal property heterogeneity is crucial when designing processing routes that result in optimum grain structure development following recrystallisation, to achieve the desired material properties.

It is shown that the effect of a lower thermal conductivity of the particles in the presence of a thermal gradient within the material is twofold; primarily the particles exhibit a shielding effect on any boundaries down the thermal gradient. The heterogeneity in thermal conductivities between the particles and grains also initiates a thermal gradient directed at the particles upon cooling, as shown in Fig. 2, this adds an additional ‘sticking’ force between the grain boundary and the particle. The net effect of the lower conductivity particles is that boundaries migrating up the thermal gradient in the vicinity of the particles require additional energy in order to traverse the particles than would be required if the thermal properties of the particles were the same as the matrix. It is clear that a finer dispersion of particles, corresponding to an increase in particle surface area leads to a greater pinning effect and therefore grain size distribution shifted towards smaller grain sizes.

The combined effect of the thermal shielding and additional thermal gradient driving force is most pronounced in a simulated microstructure experiencing a non-trivial thermal load, analogous to those experienced in many advanced manufacturing processes such as laser and electron beam welding and additive manufacturing. Here the finely dispersed pinning particles with a lower thermal conductivity have the greatest effect of pinning the grain boundaries and shielding the microstructure from the thermal load.

In summary:

- A lower thermal conductivity of the second phase particles results in the generation of additional thermal gradients
  - During cooling these gradients provide an additional driving force on the boundary toward the particles
  - During heating the thermal gradients point away from the particles and provide a repulsive driving force at the boundaries
- The lower thermal conductivity of the second phase particles creates a thermal shielding effect for the microstructure down the thermal gradient, perturbing the thermal response the microstructure experiences
- During a thermal cycle the net effect of the thermal gradient and thermal shielding, due to the particles, shifts the grain size distribution towards smaller equivalent diameters
  - It is shown that there is a considerable increase in the migration time of a boundary, if the thermal properties of the particle are heterogeneous; analogous to the boundary requiring extra energy than would be required for homogeneous thermal properties
- The shift in grain size distribution due to heterogeneous thermal properties of the second phase particles, is greater during a thermal heating and cooling cycle, than the shift in grain size distribution due to varying particle size

While the presented results are for a *Ti* alloy system, there is no loss in generality in applying the conclusions of this work to other metallurgical scenarios; such as grain boundary migration in fully austenitized heat affected zones in steel welds for example. In the future, the mechanical driving forces due to the induced thermal stresses should be considered for a complete description of thermally induced grain coarsening.

## Method

In order to predict the microstructural behaviour of a metallic system, a mathematical model based on fundamental physical driving forces is preferred^4^. In this work a multi-phase field model is used. This model incorporates the thermal gradient and local curvature driving forces into the free energy functional of the system and can accurately predict the microstructural behaviour due to complex thermal fields^2^. In this model, boundaries between fields are considered diffuse interfaces with finite width. The orientation, or phase, varies gradually over this boundary. An order parameter, ${\phi }_{N}$, is used to represent this orientation, in a system of N possible orientations. The sum of all order parameters in the domain at any given point is unity^29–31^. The final order parameter in the sequence, ${\phi }_{N}$, is used to represent the pinning particles. It is assumed that the pinning particles are static in the alloy matrix. A step function is defined to determine the number of coexisting phases as shown in Eq. 1. The number of phases coexisting at a given point is then given by Eq. 2

1 $${s}_{n}=\{\begin{array}{ll}1; & \forall {\phi }_{n} > 0\\ 0; & \forall {\phi }_{n}=0\end{array}$$

2 $$S=\mathop{\sum }\limits_{n=1}^{N}\,{s}_{n}$$

The evolution equation for the phase fields is then given by

3 $$\frac{\partial {\phi }_{n}}{\partial t}=\frac{2{M}_{\phi }}{S}\,\mathop{\sum }\limits_{p\ne n}^{N}\,{s}_{p}{s}_{n}(\frac{\delta F}{\delta {\phi }_{n}}-\frac{\delta F}{\delta {\phi }_{p}})$$

where ${M}_{\phi }$ is the isotropic phase field mobility, and has the form ${M}_{\phi }=m\sigma /{\epsilon }^{2}={\pi }^{2}m/16\,\xi$^32,33^. Here *σ* is the grain boundary energy, $\epsilon$ is the gradient energy coefficient and *m* is the grain boundary mobility and is often assumed to follow an Arrhenius relationship with temperature, *T*, of the form

4 $$m={m}_{o}\,\exp \,(\,-\frac{Q}{{k}_{B}T})$$

where *Q* is the activation energy for the grain boundary under consideration, *m*_0_ is a pre-exponential constant and *k*_*B*_ is the Boltzmann constant. The variation in the free energy density due to the grain boundary curvature and thermal gradient driving forces is given by Eq. 5

5 $$\frac{\delta F}{\delta {\phi }_{n}}=\mathop{\sum }\limits_{m\ne n}^{N}\,(\frac{{\epsilon }^{2}}{2}{\nabla }^{2}{\phi }_{m}+\omega {\phi }_{m}-\frac{\mu }{2}\nabla {\phi }_{m}\cdot \nabla T)$$

where *μ* is the temperature gradient energy coefficient of the form $\mu =16\,\xi \,2\lambda \,\Delta S/{\pi }^{2}\,\Omega$, and $\omega$ is the height of the parabolic potential with a double obstacle^3,32^. The remaining parameters are the molar volume of the material, Ω, the entropy difference between the grain boundary and bulk, Δ*S*, the phase field width, $2\,\xi$, and the grain boundary width, 2*λ*. The omission of the $-\,\frac{\mu }{2}\nabla {\phi }_{m}\cdot \nabla T$ term on the right hand side of Eq. 5 reproduces the familiar phase field expression for the evolution of a boundary network due to curvature driving forces alone^32^.

The second phase particles are assumed to be static and to not evolve in time or space. As such the *N*^*th*^ phase is designated the particle phase and the mobility between this phase and all other phases is defined to be zero. The heat equation describes the temperature evolution in the system

6 $$\rho {c}_{p}\frac{\partial T}{\partial t}=\nabla \cdot (k\nabla T)$$

where *k* is the position dependent thermal conductivity of the material, given by $$k={\phi }_{N}{k}_{particles}+(1-{\phi }_{N})$$$${k}_{Ti6Al4V}$$. $$\rho $$ and *c*_*p*_ are the mass density and specific heat capacity at constant pressure respectively. The phase field model described in Eqs. 3 and 5 are fully coupled to Eq. 6.

The parameters used in the phase field simulation are shown in Table 1. The molar volume is calculated from the density and molar mass of *Ti6Al4V*. The relative entropy difference between the boundaries and bulk in the domain, Δ*S*, is estimated from the melting enthalpy of *Ti6Al4V*. The pre-exponential factor, *m*_0_, present in Eq. 4 for the system is not known and there is very little information of this parameter for engineering alloy systems. However, the evolution behaviour of the phase field model is relatively insensitive to this parameter and so a value available from the literature is used. It is worth noting that the simulations were far more sensitive to the activation energy, *Q*, than the pre-exponential factor, *m*_0_, and so this is a reasonable assumption. The reported values of the parameters utilised in the phase field model exhibit some degree of scatter in the literature. Every effort has been taken to use representative values for the *Ti6Al4V* system under consideration.

**Table 1:** Parameters used in the phase field simulation of the alloy system.

*Parameter*	*Ti*-*6Al*-*4V*	*Particles*
$\sigma \,(kg\,{s}^{-2})$^35^	0.81	0.81
$\Omega \,(m{m}^{3}\,mo{l}^{-1})$	28.46 × 10^3^	28.46 × 10^3^
$\Delta S\,(kg\,m{m}^{2}\,{s}^{-2}\,{K}^{-1}\,mo{l}^{-1})$	3.40 × 10^8^	3.40 × 10^8^
*Q* (*eV*)^36,37^	1.0	—
2*λ* (*nm*)^38,39^	0.5	0.5
${m}_{0}\,(m{m}^{2}\,s\,k{g}^{-1})$^40^	150.0	—
$k\,(kg\,mm\,{s}^{-3}\,{K}^{-1})$	7.3 × 10^3^	0.365 × 10^3^
$\rho \,(kg\,m{m}^{-3})$	4.42 × 10^−6^	4.42 × 10^−6^
${c}_{p}\,(kg\,m{m}^{2}\,{s}^{-2}\,{K}^{-1})$	5.7 × 10^8^	5.7 × 10^8^
*T*_*Solidus*_ (*K*)	1878.0	1878.0

The multi-phase field and heat equations were solved using a finite difference scheme for the spatial components and an explicit forward Euler time integration scheme using the C++ language^34^. Inter-node communication was performed using the message passing interface. The computational domains were decomposed into 120 sub-domains on the University of Manchester’s high performance computational shared facility.

## Supplementary information

Supplementary Video 

## Data Availability

The authors declare that the data supporting the findings of this study are available from the corresponding author upon reasonable request.
